# Investigation of the mechanism of chenodeoxycholic acid in treating acute lung injury through network pharmacology and experimental validation

**DOI:** 10.1038/s41598-025-90155-4

**Published:** 2025-02-17

**Authors:** Chong He, Mengmeng Jiang, Qian Xiong, Zuoxi Huang

**Affiliations:** 1 Department of Emergency, The First Affiliated Hospital, Jiangxi Medical College, Nanchang University, Nanchang, Jiangxi China; 2 Department of Gastrointestinal Surgery, Jiangxi Provincial People’s Hospital, The First Affiliated Hospital of Nanchang Medical College, Nanchang, 330006 Jiangxi China

**Keywords:** Chenodeoxycholic acid, Acute lung injury, Network pharmacology, Molecular docking, Animal experiment, Pharmacology, Experimental models of disease

## Abstract

Network pharmacology and molecular simulation techniques were employed to predict the potential targets and signaling pathways of chenodeoxycholic acid in the treatment of acute lung injury. Subsequently, its therapeutic effects on acute lung injury were preliminarily validated using animal experiments. The target of Chenodeoxycholic acid in the treatment of acute lung injury was predicted using network pharmacology. Key active ingredients and core targets were further validated using molecular docking studies. Lipopolysaccharide was used to establish a mouse model of acute lung injury to study the effect of chenodeoxycholic acid on acute lung injury. A total of 73 potential targets of Chenodeoxycholic acid for the treatment of acute lung injury were identified, primarily HSP90AA1, STAT3, HSP90AB1, EP300, and NFKB1. These core targets influence pathways associated with bile secretion, prostate cancer, and receptor activation in chemical carcinogenesis. These targets modulate various processes, including steroid metabolism, steroid biosynthesis, and intracellular receptor signaling pathways, thus contributing to the treatment of acute lung injury. Molecular docking results indicated that Chenodeoxycholic acid exhibited strong binding affinity for the core targets, with docking energies ranging from −5.6729 to −7.4138 kcal/mol. The reliability of the results was further verified by molecular dynamics simulations. Results from animal experiments demonstrated that Chenodeoxycholic acid effectively ameliorated pathological injury to lung tissue in mice with acute lung injury, decreased levels of IL-6 and TNF-α (P < 0.01), and increased levels of IL-10 (P < 0.01). The mRNA expression levels of EP300, HSP90AB1, MTOR, and STAT3 were inhibited, while the mRNA expression level of NR1H4 was significantly increased (P < 0.01). Chenodeoxycholic acid can effectively improve acute lung injury.

## Introduction

Acute lung injury (ALI) is clinically characterized by acute respiratory insufficiency, accompanied by shortness of breath, hypoxemia, reduced pulmonary compliance, and diffuse alveolar infiltration visible on chest radiographs. Without timely clinical intervention, ALI can progress to acute respiratory distress syndrome, which has a mortality rate as high as 40–60%^1–3^. Mechanical ventilation and glucocorticoids are currently the primary treatments for ALI; however, they have significant limitations. While mechanical ventilation is essential for maintaining adequate oxygenation, it can exacerbate the underlying ALI through ventilator-induced lung injury (VILI), particularly when high pressure or volume settings are employed. Additionally, mechanical ventilation increases the risk of infections, such as ventilator-associated pneumonia, due to the insertion of breathing tubes and suppression of the immune system. These infections may further deteriorate the patient’s condition. Prolonged use of mechanical ventilation is associated with complications such as muscle weakness, dysphagia, and an elevated risk of chronic lung disease. Although glucocorticoids are effective in reducing inflammation, they are associated with significant side effects, including hyperglycemia, osteoporosis, and immunosuppression. These adverse effects are particularly severe for critically ill ALI patients. Moreover, glucocorticoids may exhibit variable efficacy in mitigating inflammation depending on the stage and severity of the injury. The optimal timing and dosage of glucocorticoids for ALI treatment remain unclear, with improper dosing—whether excessive or insufficient—potentially rendering the treatment ineffective or harmful. In view of these limitations, it is imperative to explore novel therapeutic agents for the treatment of ALI. These agents should be both safe and effective by targeting the underlying pathophysiological mechanisms of the injury and minimizing adverse effects.

Bile acids (BAs) are synthesized by the liver and serve as physiological cleansers that promote bile flow and absorption in the intestine, facilitating the transport of nutrients, vitamins, and lipids while participating in multifunctional signaling processes. The Farnesoid X receptor (FXR) is a nuclear receptor activated by bile acids that regulates numerous physiological and pathological functions. Bile acids, as ligands of the Farnesoid X receptor (FXR), exert broader biological effects^4^. Studies have shown that bile acids inhibit the activation of the NLRP3 inflammasome via the TGR5-CAMP-PKA axis, where activation of PKA induced by TGR5 bile acid receptors leads to the ubiquitination of NLRP3^5^.

Chenodeoxycholic acid (CDCA) is a principal component of animal bile and is classified as a bile acid. CDCA regulates cholesterol metabolism and is utilized in the treatment of various liver and biliary diseases, such as cholestasis, cholesterol stones, and stasis liver disease. CDCA also exhibits anti-inflammatory and protective effects on intestinal barrier function. Additionally, CDCA demonstrates significant effects on antioxidant activity, anti-inflammatory responses, and immune regulation^6–10^. Zhu et al.^11^ demonstrated that CDCA and its derivative, obeticholic acid, exhibited protective effects against acinar cell injury and pancreatic necrosis in a mouse model, with CDCA showing notable anti-inflammatory properties. Although numerous studies have elucidated the various biological functions of CDCA, the mechanism underlying its action in the treatment of acute lung injury remains to be fully explored. ALI is an inflammatory response in the lung caused by a variety of factors, leading to increased pulmonary capillary permeability and pulmonary edema. Given the good anti-inflammatory effect of CDCA, we hypothesized that CDCA could reduce pulmonary inflammation and edema by inhibiting inflammatory response and cytokine release, thereby improving respiratory function and prognosis of patients.

Network pharmacology research effectively facilitates the in-depth exploration of the biological activity of drugs and clarifies the molecular mechanisms underlying drug treatment of diseases^12^. The traditional treatment of ALI often targets individual pathways or mechanisms, such as anti-inflammatory effects, antioxidant responses, or improved ventilation strategies. However, CDCA identified through network pharmacology as a promising therapeutic agent, may act on multiple biological processes implicated in the pathogenesis of ALI by targeting multi-pathway mechanisms. This represents a novel treatment concept. ALI is a complex disease involving disruptions in numerous biological processes. By targeting these multipathway mechanisms, CDCA can more comprehensively address the underlying disturbances, offering the potential for improved therapeutic outcomes. Therefore, this study identified the core targets and associated signaling pathways of CDCA in the treatment of ALI through network pharmacology and established an LPS-induced mouse model of ALI to further investigate the specific therapeutic mechanisms of CDCA on ALI.

## Materials and methods

### Databases and materials

The following databases were utilized in this study: the PubChem database (https://pubchem.ncbi.nlm.nih.gov), UniProt database (https://www.uniprot.org), GeneCards database (https://www.genecards.org), BATMAN-TCM database (http://bionet.ncpsb.org/batman-tcm/), and DAVID database (https://david.ncifcrf.gov/). Network analysis was performed using the STRING online analysis platform (https://string-db.org), and Cytoscape software was employed for visualization.

Chenodeoxycholic acid was procured from Jiangyin Tianjiang Pharmaceutical Co., Ltd. (Jiangsu, China). Lipopolysaccharide was obtained from Solaibao Technology Co., Ltd. (Beijing, China), and isoflurane was supplied by Rayward Life Technology Co., Ltd. (Shenzhen, China). Mouse IL-6, IL-10, and TNF-α ELISA kits were purchased from Nanjing Jiancheng Biotechnology Co., Ltd. Primers for qRT-PCR were synthesized by Shanghai Shenggong Bioengineering Co., Ltd.

### Network pharmacology and molecular docking

#### CDCA targets, ALI targets and their common targets were acquired

CDCA was searched in the PubChem database, and its SMILES notation was copied and input into the SwissTargetPrediction website to retrieve potential targets. The UniProt database was then used to standardize the targets and obtain genetic information. The InChI key of chenodeoxycholic acid was entered into the BATMAN-TCM database to predict its targets. Using “ALI” as a keyword, GeneCards was searched to identify relevant ALI-related targets. Venny software was used to identify the intersection between chenodeoxycholic acid targets and ALI-related targets, determining the potential targets of chenodeoxycholic acid for the treatment of ALI^13^.

#### Construction of potential target PPI network and screening of core targets

The common target genes of chenodeoxycholic acid and ALI were uploaded to the STRING online analysis platform to construct the Protein–Protein Interaction (PPI) network. Species was set to Homo sapiens.Minimum required interaction score was set to 0.9 (high confidence) to ensure result reliability. The results were stored in TSV format, imported into Cytoscape 3.9.2, and the network was analyzed. The degree values were visualized using Cytoscape software, with size and color changes to display the network^14^.

#### GO and KEGG pathway enrichment analysis of core target genes

The intersecting genes were input into the DAVID database for Gene Ontology (GO) functional enrichment analysis and Kyoto Encyclopedia of Genes and Genomes (KEGG) pathway enrichment analysis. Pathways and GO terms with a P-value < 0.01 were selected from the enriched items, and the top 30 pathways and top 10 GO terms based on count values were selected for visualization^15^.

#### “CDCA-ALI-pathway” network construction

The common targets of chenodeoxycholic acid and ALI, along with the main pathways identified through KEGG pathway enrichment analysis, were imported into Cytoscape 3.8.0 software to construct the “CDCA-ALI-pathway” network^16^.

#### Molecular docking

Molecular docking was performed according to the core targets screened by network pharmacology. The 2D structure of CDCA (ID:10133) was retrieved as a small molecule ligand from the PubChem database (http://pubchem.ncbi.nlm.nih.gov/) and then input into ChemOffice software to generate a 3D structure, which was saved as a mol2 file. The RCSB PDB database was used to screen the protein targets, and the crystal structure with the highest available resolution was selected as the molecular docking receptor. Selecting core targets as molecular docking receptors, They were HSP90AA1(ID:5LQ9), NFKB1(ID:1SVC), STAT3(ID:6NUQ), CD4(ID:3S5L), HSP90AB1(5UCI), NR1H4(ID:4WVD), EP300(ID:6V90), MTOR(ID:5ZCS), TLR4(ID:3FXI). PyMOL software was used to remove water and phosphate groups from the protein, and the processed structure was saved as a PDB file. Molecular Operating Environment (MOE) 2019 software was employed to minimize the energy of the compounds, preprocess the target proteins, and identify the active sites. Finally, molecular docking was performed using MOE 2019. The binding energies were evaluated to assess the affinity between the ligand and receptor, and the results were visualized using PyMOL and Discovery Studio software^17^.

#### Molecular dynamics

The three complexes with the best binding energies in molecular docking were selected for molecular dynamics (MD) simulations to enhance the reliability of the results. The complexes were subjected to 50-ns MD simulations using Gromacs 2023. The CHARMM36 force field parameters were applied to the protein, and the ligand topology was generated using the GAFF2 force field. Periodic boundary conditions were applied, with the protein–ligand complex positioned within a cubic simulation box. The TIP3P water model was employed to solvate the simulation box with water molecules. Electrostatic interactions were calculated using the particle mesh Ewald (PME) method and the Verlet algorithm. Subsequently, the system underwent NVT (isothermal-isochoric) and NPT (isothermal-isobaric) ensemble equilibrations, with a coupling constant of 0.1 ps over a 100-ps duration. Both van der Waals and Coulomb interactions were computed using a cutoff of 1.0 nm. Finally, a 50-ns MD simulation was conducted using Gromacs 2023, with the system maintained at a constant temperature of 300 K and pressure of 1 bar^18^. The operating system is Ubuntu 18.04, equipped with Python 3.8, providing a stable development and computing environment. CUDA 11.4 supports GPU acceleration, which significantly improves the computing performance. In terms of hardware, the system is equipped with an RTX 2080 Ti graphics card with 11GB of video memory, which can accelerate complex calculations in large-scale molecular dynamics simulations. The CPU is an Intel(R) Xeon(R) Platinum 8255C with 12 cores at 2.50GHz.

### Animal experiments

#### Experimental animals and grouping

The study is in accordance with the ARRIVE guidelines. Fifty SPF male BALB/c mice, aged 4–6 weeks and weighing 20 g, were purchased from the Experimental Animal Center of Nanchang University. In this study, the mice were randomly divided into five groups: normal group, model group, low-dose group, medium-dose group, and high-dose group of Chenodeoxycholic acid, with ten mice in each group, following one week of adaptive feeding approved by the Experimental Animal Ethics Committee of the First Affiliated Hospital of Nanchang University (SYXK2021-003). The experimental procedures were conducted in accordance with applicable guidelines and regulations, as outlined in the ARRIVE guidelines. Following the modeling method established by Ye et al.^19^, the mice were anesthetized with isoflurane and administered a 10 mg/kg lipopolysaccharide solution via nasal drip, while the normal group received nasal saline. Based on previous studies and pre-experimental results^20^, the doses for the low, medium, and high groups were set at 30, 60, and 90 mg/kg, respectively, while the control and model groups were administered the same volume of normal saline for seven consecutive days. Following drug administration, the cervical vertebrae of the mice were dislocated after blood was collected from the eye; serum was then used for ELISA detection, while lung tissue was utilized for pathological observation and qRT-PCR analysis. All procedures involving the mice were performed under respiratory anesthesia using a mixture of isoflurane and oxygen to alleviate pain. Upon completion of the experiments, the mice were euthanized by CO_2_ inhalation to collect tissues for analysis.

#### Hematoxylin eosin staining of lung tissue

The lung tissue, fixed with 4% paraformaldehyde, was embedded in paraffin wax and sectioned to a thickness of 5 μm. Following HE staining, the lung histopathological changes were observed under a light microscope.

#### Serum inflammatory factors detection by ELISA

Following blood collection, the blood was centrifuged at 3,000 r/min for 20 min; serum was then separated into a sterile EP tube and stored in a refrigerator at −80 °C. Once the kit was equilibrated to room temperature, the sample, enzyme-labeled antibody, chromogenic developer, and termination solution were added sequentially according to the kit specifications. Absorbance was measured at a wavelength of 450 nm, and the concentrations of IL-6, IL-10, and TNF-α were calculated.

#### RT-qPCR was used to detect mRNA expression

The RT-qPCR method was employed for detection; total RNA was extracted using an RNA extraction kit, and cDNA was reverse-transcribed according to the manufacturer’s instructions. PCR amplification was subsequently performed. The PCR amplification conditions were pre-denatured at 95 ℃ for 30 s, denatured at 95 ℃ for 5 s, and annealed at 60 ℃ for 30 s for a total of 40 cycles. Using GAPDH as a reference gene, the expression levels of iNOS and COX-2 in cells were calculated using the 2^-ΔΔC^ method. The target gene primers were designed and synthesized by Shenggong Bioengineering (Shanghai) Co., Ltd. The primer sequences and product sizes are presented in Table 1.

**Table 1:** The primer sequences used for RT-qPCR.

Primer	Primer sequence(5′-3′)
EP300	F: ACCTTCTCCTGTTCCTAGCCGTAC R: AATTGCTGTTGCTGCTGGTTGTTG
HSP90AB1	F: CATCTCCATGATTGGGCAGTT R: CTTTGACCCGCCTCTCTTCTA
mTOR	F:CAGTTCGCCAGTGGACTGAAG R:TTGTAGCCAATAAAGGTGCCAT
STAT3	F: CAATACCATTGACCTGCCGAT R: GAGCGACTCAAACTGCCCT
NR1H4	F:GACCACGAAGACCAGATTGC R:GAGATGCCGCTCTTTCGAAT
GAPDH	F: CCTCGTCCCGTAGACAAAATG R: TGAGGTCAATGAAGGGGTCGT

### Statistical analysis

Excel, Spss 26, and GraphPad Prism 8 software were used to process and plot the data. Consistent with normal distribution and homogeneity of variance, One-Way ANOVA was used for comparisons between multiple groups with a single independent variable. P < 0.05 indicated that the difference was statistically significant. A P value of less than 0.01 was considered highly significant.

## Results

### Screening of common targets of CDCA and ALI

**Fig. 1:**
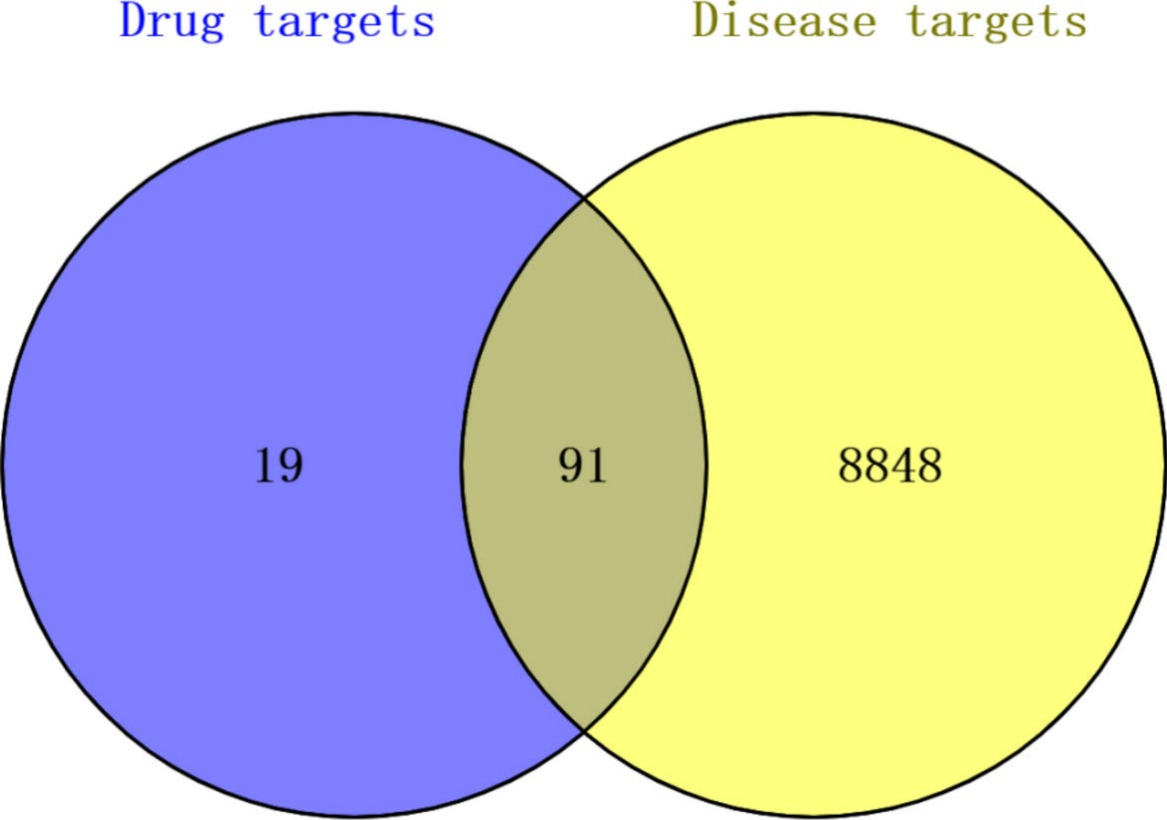
Venn diagram of common target of CDCA and ALI.

Figure 1 presents a Venn diagram illustrating the common targets of CDCA and ALI. CDCA was found to have 110 potential targets, whereas ALI was associated with 8,939 targets. The target genes related to both CDCA and ALI were screened using a bioinformatics analysis and visualization platform, resulting in the identification of 91 common target genes.

### Potential targets for PPI network building and core targets selection

**Fig. 2:**
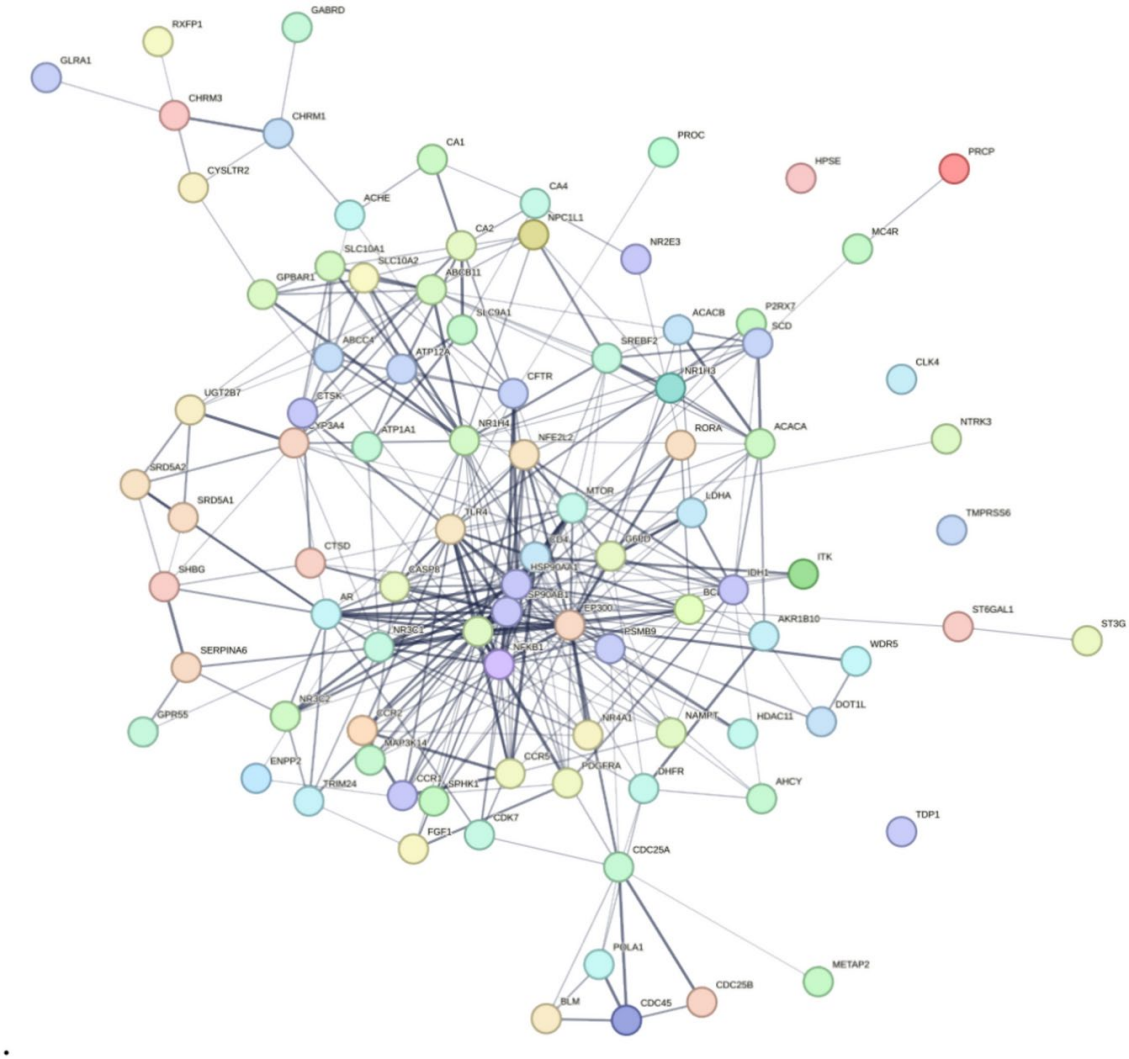
PPI network of CDCA and ALI intersection genes.

**Fig. 3:**
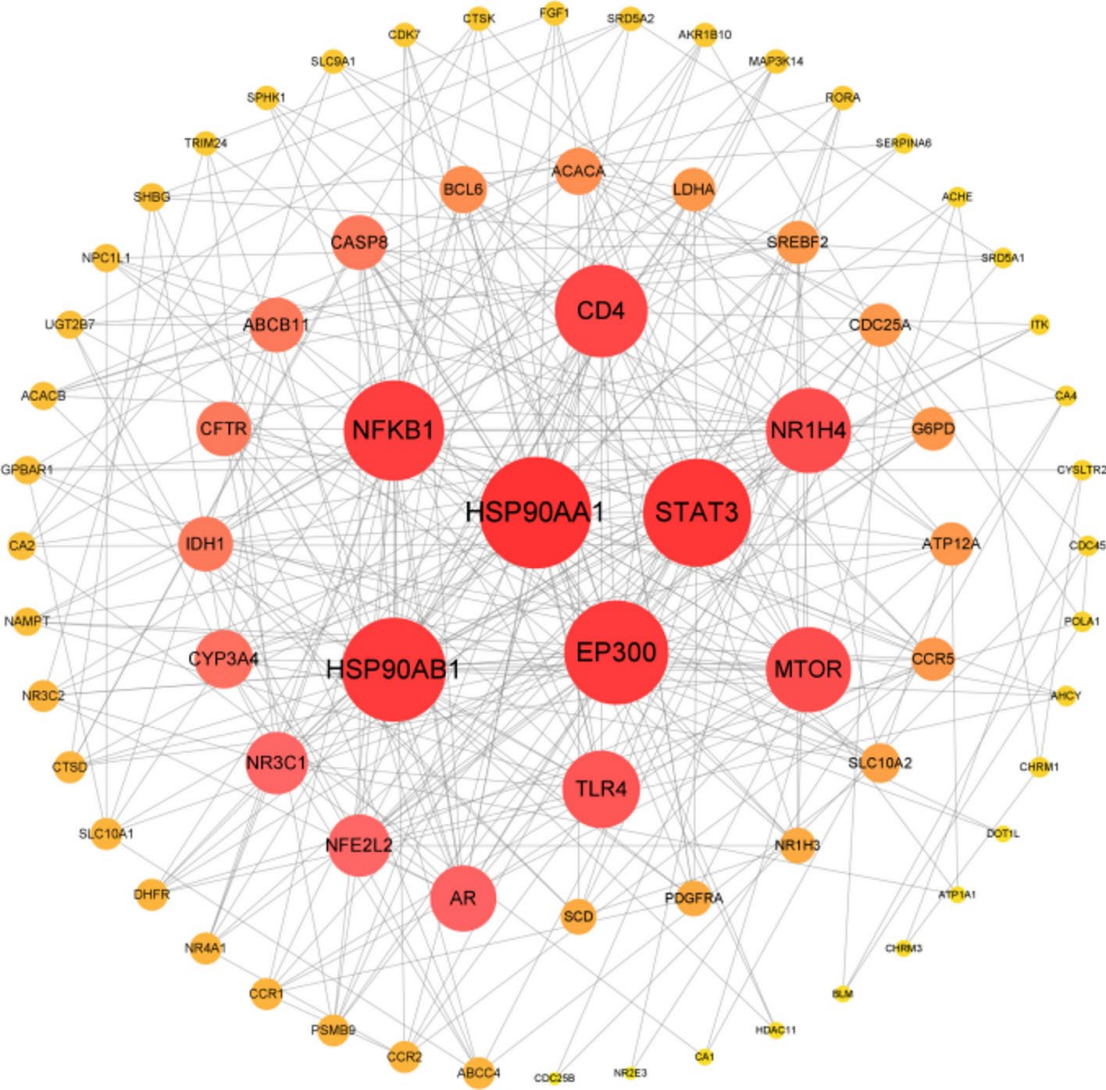
PPI network of key genes of CDCA and ALI intersection.

The PPI network topology was analyzed using the CytoNCA plugin, and the core targets were screened based on their degree value. Figure 2 displays the PPI network of intersecting genes between CDCA and ALI, while Fig. 3 presents the PPI network of the critical genes. Figure 3 identifies the nine core targets of CDCA in the treatment of ALI as HSP90AA1, STAT3, HSP90AB1, EP300, NFKB1, CD4, NR1H4, MTOR, and TLR4.

### The core GO and KEGG pathway enrichment analysis of target genes

**Fig. 4:**
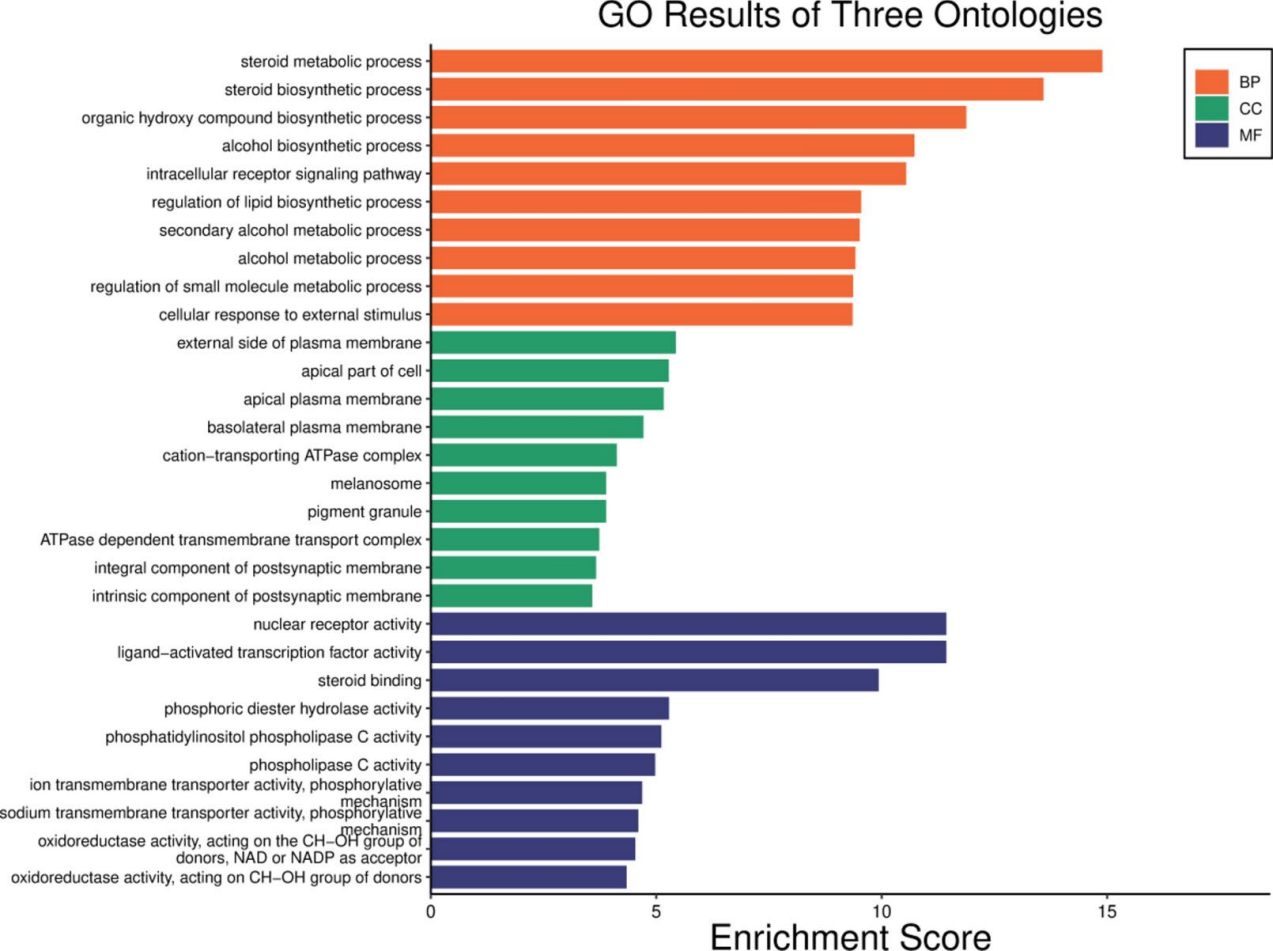
GO enrichment map of potential targets of CDCA in the treatment of ALI.

Figure 4 illustrates the Gene Ontology (GO) enrichment results across three ontologies: Biological Processes (BP), Cellular Components (CC), and Molecular Functions (MF). The x-axis represents the enrichment score, which indicates the degree of over-representation of the GO terms in the dataset, while the y-axis lists the GO terms. In the Biological Processes (BP) category, the steroid metabolic process exhibits the highest enrichment score, indicating significant over-representation. This is followed closely by the steroid biosynthetic process, suggesting a focus on steroidogenesis. Furthermore, such as the organic hydroxy compound biosynthetic process, alcohol biosynthetic process, intracellular receptor signaling pathway, regulation of lipid biosynthetic process, secondary alcohol metabolic process, alcohol metabolic process, regulation of small molecule metabolic process, and cellular response to external stimuli are also significantly enriched.

For Cellular Components (CC), the enriched terms include the extrinsic component of the plasma membrane, apical part of the cell, basolateral plasma membrane, plasma membrane, cation-transporting ATPase complex, melanosome, and pigment granule. These results indicate a strong representation of plasma membrane components and regions involved in various cellular functions.

In the Molecular Functions (MF) category, significant enrichment is observed in activities such as ATPase-dependent transmembrane transporter activity, integral and intrinsic components of the postsynaptic membrane, nuclear receptor activity, ligand-activated transcription factor activity, steroid binding, phosphoric diester hydrolase activity, phosphatidylinositol phospholipase C activity, phospholipase C activity, sodium transmembrane transporter activity via a phosphorylative mechanism, oxidoreductase activity, and oxidoreductase activity acting on CH-CH groups with NAD or NADP as acceptors.

The results highlight significant enrichment in processes related to steroid metabolism and biosynthesis, regulation of lipid biosynthesis, and alcohol metabolism within the Biological Processes ontology. For Cellular Components, there is a strong representation of plasma membrane components and regions, while Molecular Functions highlight activities related to ATPase-dependent transport, nuclear receptor activity, and oxidoreductase functions. These enriched GO results provide insights into the molecular functions, cellular components, and biological processes that are significantly represented in the dataset, suggesting focus areas for further experimental investigation.

**Fig. 5:**
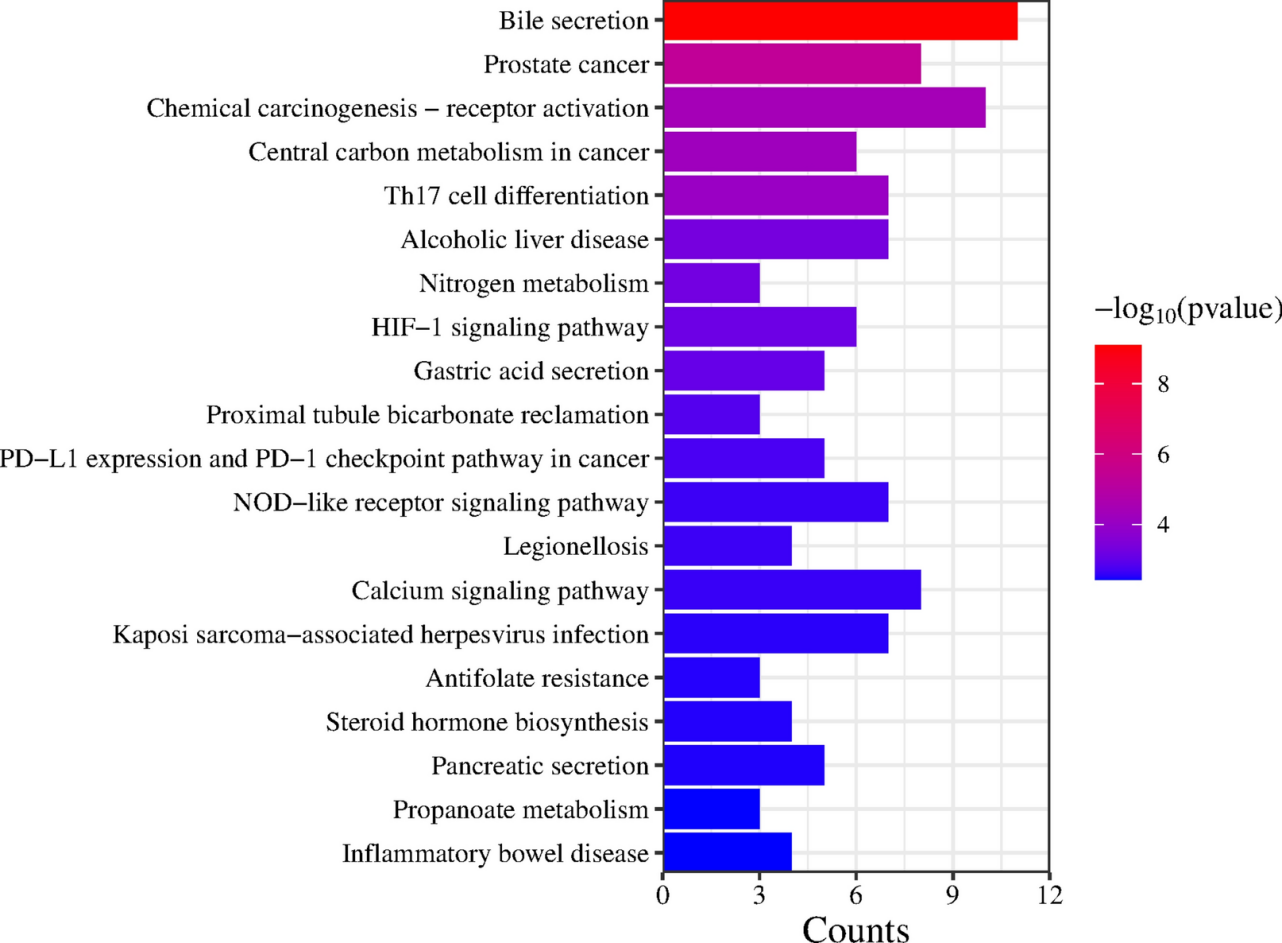
KEGG pathway enrichment map of potential targets of CDCA in the treatment of ALI. The identified pathways include Bile secretion, Prostate cancer, Chemical carcinogenesis–receptor activation, Central carbon metabolism in cancer, Th17 cell differentiation, Alcoholic liver disease, Nitrogen metabolism, HIF-1 signaling pathway, Gastric acid secretion, Proximal tubule bicarbonate reclamation, PD-L1 expression and PD-1 checkpoint pathway in cancer, NOD-like receptor signaling pathway, Legionellosis, Calcium signaling pathway, Kaposi sarcoma-associated herpesvirus infection, Antifolate resistance, Steroid hormone biosynthesis, Pancreatic secretion, Propanoate metabolism, and Inflammatory bowel disease. Pathway significance is represented by -log10 (p-value), with a greater intensity of red indicating higher significance. The horizontal axis reflects the target count in each pathway.

Figure 5 illustrates the results of the KEGG pathway enrichment analysis^21^. Key pathways include bile secretion, which has the highest gene count and is the most statistically significant. This suggests a strong involvement of the bile secretion pathway in the dataset, possibly indicating a role in metabolic regulation or detoxification processes. The prostate cancer pathway follows, with the second highest gene count and significant statistical relevance, indicating its involvement in cancer-related processes and pathways associated with cellular proliferation and differentiation. The chemical carcinogenesis-receptor activation pathway also exhibits a high gene count and statistical significance, suggesting its involvement in pathways related to chemical-induced carcinogenesis and potentially highlighting receptor-mediated processes. The central carbon metabolism in cancer pathway shows a moderate gene count and significant enrichment, underscoring pathways involved in metabolic reprogramming in cancer cells. Similarly, Th17 cell differentiation demonstrates a moderate gene count and significant enrichment, indicating its role in immune response and regulation of inflammation. The alcoholic liver disease pathway is also significant, suggesting involvement in liver disease, potentially highlighting metabolic and oxidative stress responses.

Other notable pathways include nitrogen metabolism, HIF-1 signaling, gastric acid secretion, proximal tubule bicarbonate reclamation, PD-L1 expression and PD-1 checkpoint regulation in cancer, NOD-like receptor signaling, legionellosis, calcium signaling, Kaposi sarcoma-associated herpesvirus infection, antifolate resistance, steroid hormone biosynthesis, pancreatic secretion, propanoate metabolism, and inflammatory bowel disease. These pathways encompass various processes, including nitrogen cycling, cellular responses to hypoxia and stress, digestive processes, kidney function, immune checkpoint regulation, innate immune responses, muscle contraction, neurotransmitter release, viral infections, antifolate drug resistance, steroid hormone production, and chronic inflammatory conditions of the gut. The KEGG pathway enrichment analysis reveals significant involvement of pathways related to bile secretion, cancer metabolism, chemical carcinogenesis, immune response, and metabolic processes. The highly significant pathways suggest potential areas of focus for understanding the molecular mechanisms underlying the studied condition or treatment.

### Molecular docking results

**Fig. 6:**
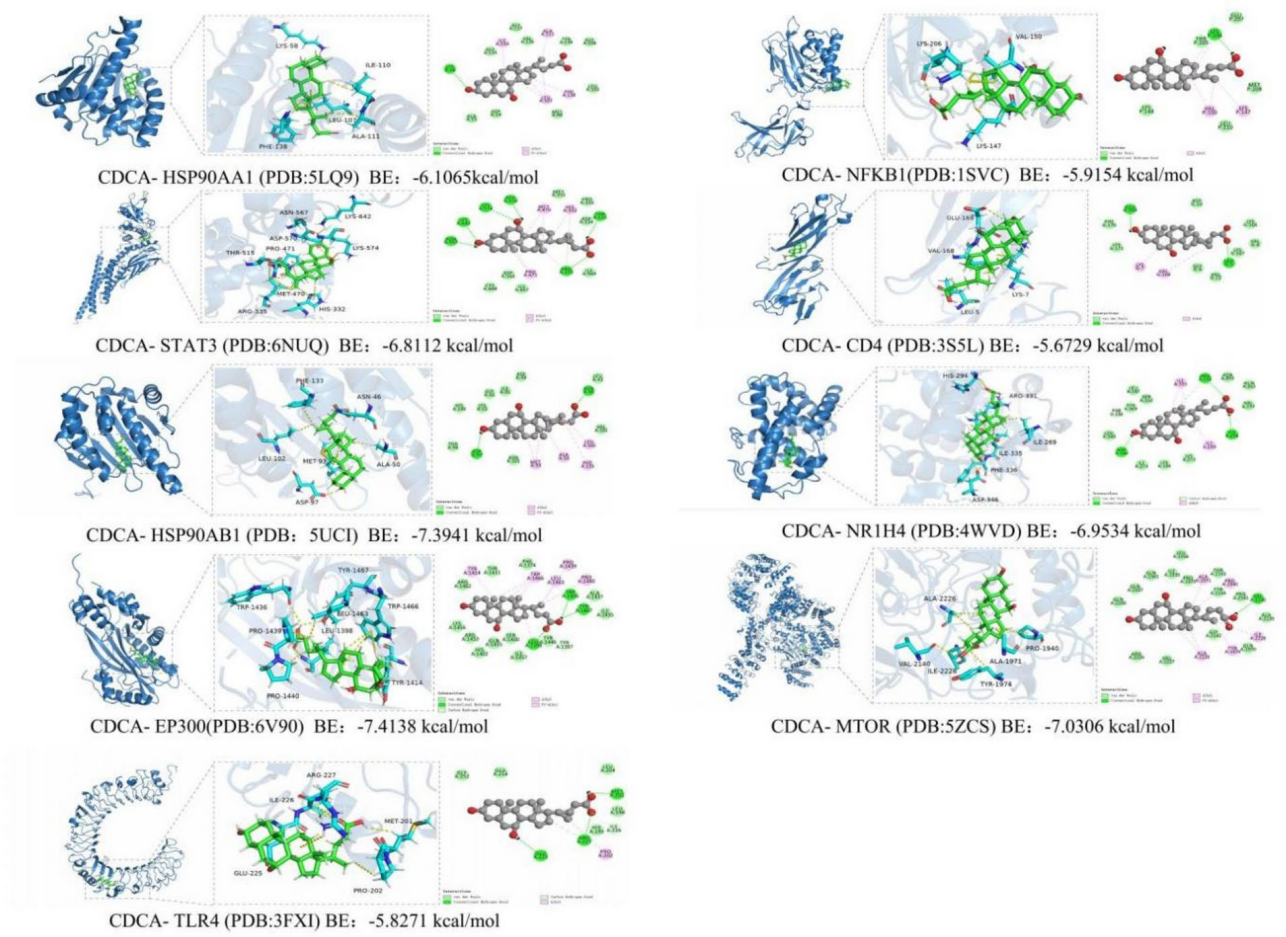
CDCA and core target protein molecular docking results.

**Fig. 7:**
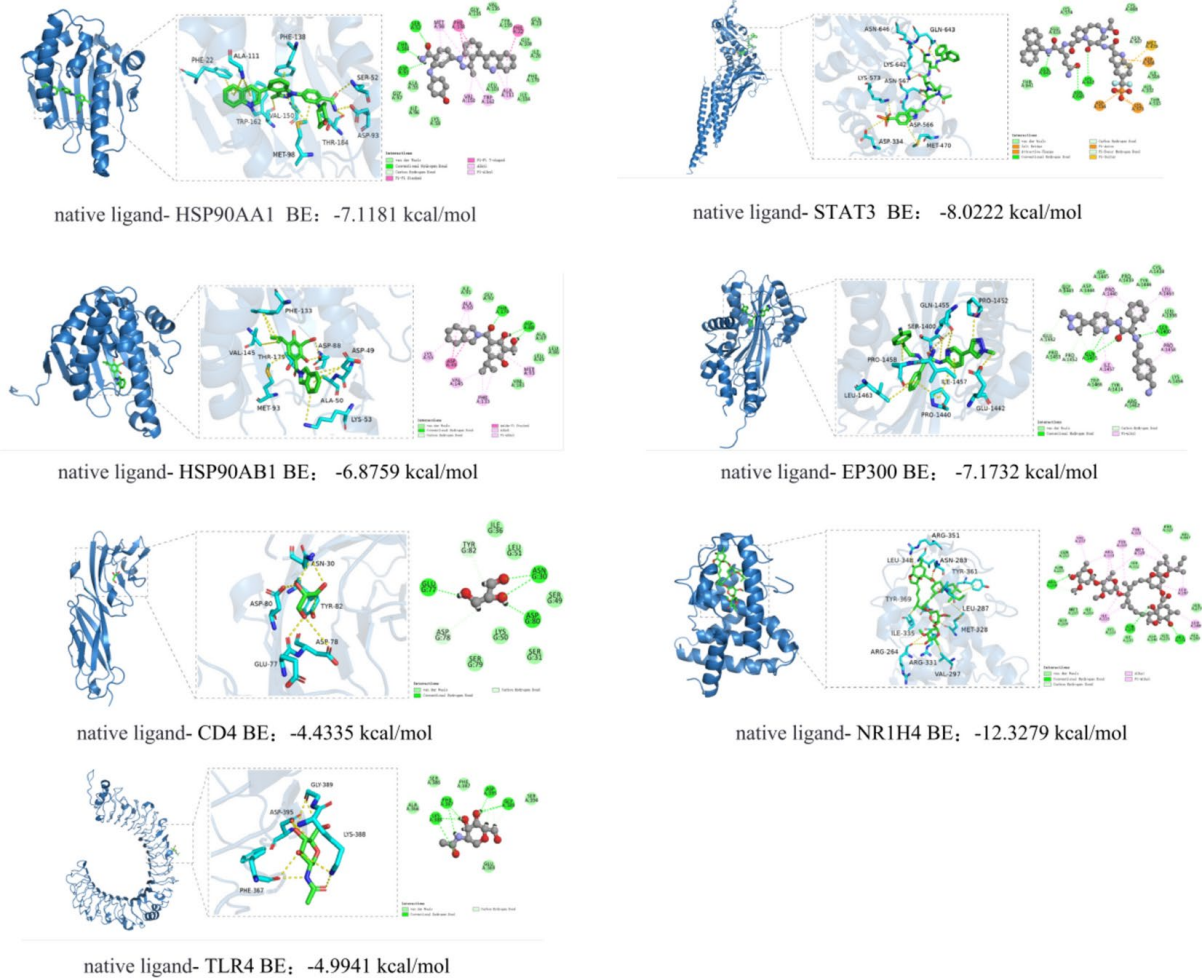
Molecular docking results of the native ligand and the corresponding target.

Figure 6 presents the molecular docking results, illustrating the interactions between CDCA and core target proteins as analyzed using MOE 2019 software. The binding energy and binding site analysis of CDCA to the corresponding targets are detailed in Table 2. The results indicate that CDCA interacts with nine target protein domains. The docking energy between CDCA and the target proteins ranged from −5.6729 to −7.4138 kcal/mol. Studies indicate that docking energies below −4.25 kcal/mol suggest a specific interaction, below -5.0 kcal/mol indicate good binding activity, and below -−.0 kcal/mol signify a highly potent interaction. The most favorable interaction was observed between CDCA and EP300, with a docking energy of −7.4138 kcal/mol, followed by CDCA-HSP90AB1 (−7.3941 kcal/mol), CDCA-MTOR (−7.0306 kcal/mol), and CDCA-NR1H4 (−6.9534 kcal/mol).The Lys58 residue on the HSP90AA1 receptor forms a hydrogen bond with CDCA, while the residues Ile110, Ala111, Leu107, and Phe138 form hydrophobic interactions. The residues Asn567, Lys642, Lys574, Asp570, Thr515, and Arg335 on the STAT3 receptor form hydrogen bonds with CDCA, and the residues Met470, His332, Pro471, and Arg335 on STAT3, along with CDCA, form hydrophobic interactions. On the HSP90AB1 receptor, the residues Asp97 and Asn46 form hydrogen bonds with CDCA, and the residues Met93, Ala50, Phe133, and Leu102 on HSP90AB1, along with CDCA, form hydrophobic interactions. The EP300 receptor residues Trp1436, Tyr1467, and Leu1398 form hydrogen bonds with CDCA, while Tyr1414, Trp1466, Leu1463, Pro1439, Pro1440, and Leu1398, along with CDCA, form hydrophobic interactions. The residue Lys206 on the NFKB1 receptor forms a hydrogen bond with CDCA, and the residues Val150, Lys147, and Lys206 in NFKB1 form hydrophobic interactions with CDCA. On the CD4 receptor, the residues Glu169 and Leu5 form hydrogen bonds with CDCA, and Lys7 and Val168 form hydrophobic interactions with CDCA. The residues Arg331, His294, and Asp346 on the NR1H4 receptor form hydrogen bonds with CDCA, while Ile335, Ile269, and Asp331, along with CDCA, form hydrophobic interactions. The residues Ala2226, Tyr1974, Ile2228, Ala1971, and Pro1940 on the MTOR receptor, along with CDCA, form hydrophobic interactions. On the TLR4 receptor, the residues Glu225, Arg227, and Met201 form hydrogen bonds with CDCA, while the residues Pro202 and Arg227 form hydrophobic interactions with CDCA. Additionally, the residue Ile226 forms a C-H interaction with CDCA. By comparing the binding energy between CDCA and the protoligand, we found that CDCA exhibits comparable or stronger binding affinity to the target. The results are presented in Fig. 7. The binding energies and binding sites of the natural ligands to the corresponding targets are analyzed in Table 3. Hydrogen bonds are crucial in stabilizing the formation of complexes between ligands and receptors. The interaction of multiple hydrogen bonds significantly influences the overall binding affinity. Hydrophobic interactions also play a pivotal role in stabilizing the complex, often involving residues located within the binding pocket. Certain ligands exhibit additional C-H interactions. These findings underscore the potential of CDCA in interacting with target proteins, suggesting its promise as a candidate for ALI treatment, warranting further investigation in drug discovery and biochemical studies.

**Table 2:** Binding energy and binding site analysis of CDCA and corresponding targets.

	Target	Binding energy of CDCA to the target site	Hydrogen bond interactions	Hydrophobic interactions	Hydrocarbon interaction
1	HSP90AA1	−6.1065	Lys58	Ile110、Ala111、Leu107、Phe138	
2	STAT3	−6.8112	Asn567、Lys642、Lys574、Asp570、Thr515、Arg335	Met470、His332、Pro471、Arg335	
3	HSP90AB1	−7.3941	Asp97、Asn46	Met93、Ala50、Phe133、Leu102	
4	EP300	−7.4138	Trp1436、Tyr1467、Leu1398	Tyr1414、Trp1466、Leu1463、Pro1439、Pro1440、Leu1398	
5	NFKB1	−5.9154	Lys206	Val150、Lys147、Lys206	
6	CD4	−5.6729	Glu169、Leu5	Lys7、Val168	
7	NR1H4	−6.9534	Arg331、His294、Asp346	Ile335、Ile269以及Asp331	
8	MTOR	−7.0306	Ala2226、Tyr1974、Ile2228、Ala1971、Pro1940		
9	TLR4	−5.8271	Glu225、Arg227、Met201	Pro202、Arg227	Ile226

**Table 3:** Binding energy and binding site analysis of native ligand and corresponding targets.

	Target	Binding energy of native ligand to the target site	Hydrogen bond interactions	Hydrophobic interactions	Hydrocarbon interaction
1	HSP90AA1	−7.1181	Ser52、Thr184、Asp93	Met98、Phe138、Phe22、Ala111、Trp162、Val150	
2	STAT3	−8.0222	Gln643、Asn646、Lys642	Asp566、Lys573、Asp334	
3	HSP90AB1	−6.8759	Asp88、Thr179	Ala50、Lys53、Asp49、Val145、Phe133、Met93	
4	EP300	−7.1732	Gln1455、Ser1400	Pro1440、Leu1463、Pro1458、Ile1457	Glu1442、Pro1452
5	CD4	−4.4335	Glu77、Asp80、Asn30		Tyr82、Asp78
6	NR1H4	−12.3279	Arg264、Asn283、Arg351	Val297、Arg331、Tyr369、Met328、Tyr361、Leu287、Leu348、Ile335	
7	TLR4	−4.9941	Lys388、Phe367、Asp395、Gly389		Phe367、Asp395、Gly389

### Molecular dynamics simulation results

**Fig. 8:**
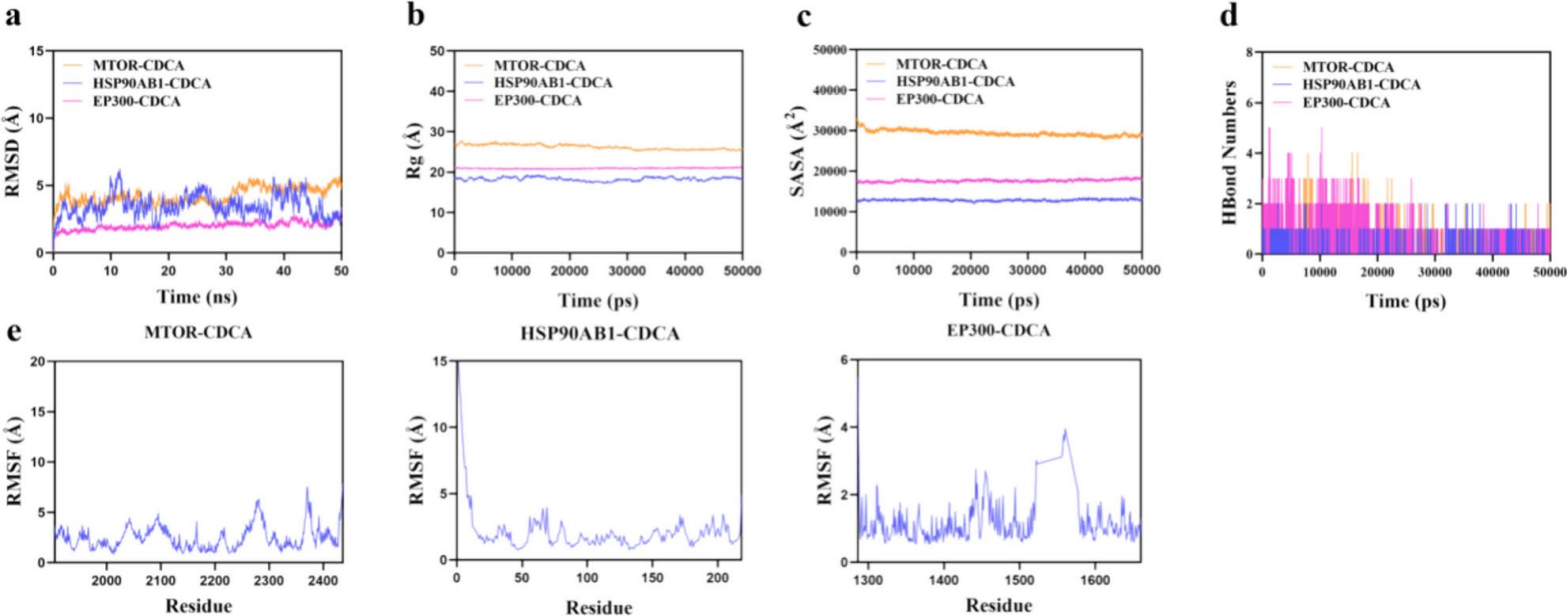
Molecular dynamics results of the native ligand and the corresponding target.

**Fig. 9:**
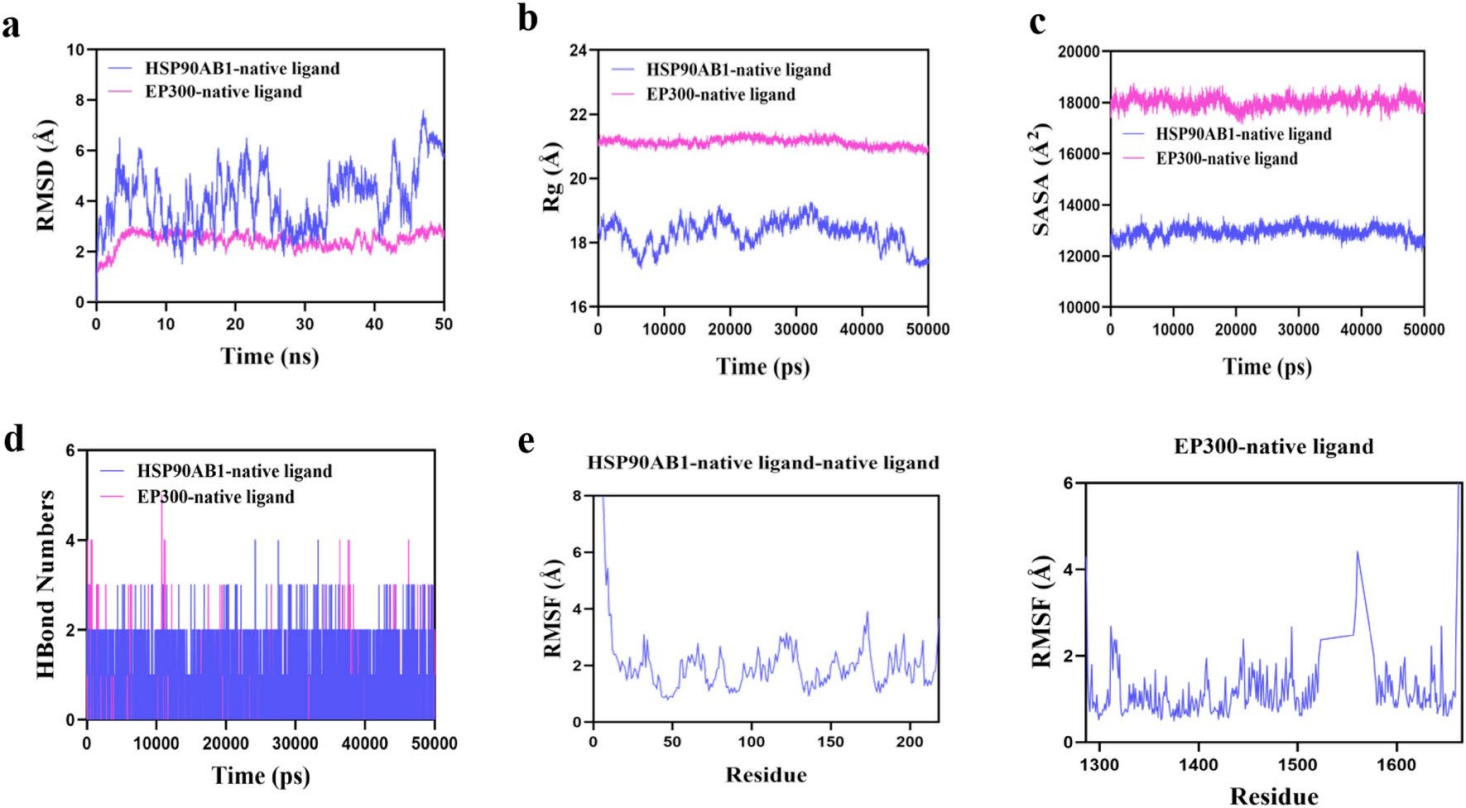
Molecular dynamics simulation results of CDCA and core target proteins.

The root mean square deviation (RMSD) is an effective measure of the conformational stability of proteins and ligands, quantifying the degree of atomic positional deviation from the initial configuration. Smaller deviations indicate greater conformational stability. Thus, RMSD is utilized to assess the equilibrium of the simulation system. Molecular dynamics results for the core target and its original ligand are provided in Fig. 8. The results show that the HSP90AB1-native ligand complex reaches equilibrium after 45 ns and stabilizes at approximately 6.6 Å. The EP300-native ligand complex reaches equilibrium after 5 ns and stabilizes around 2.8 Å. However, compared to the native ligand, CDCA demonstrated more stable binding with various target proteins. The MTOR-CDCA complex system reached equilibrium at 35 ns and stabilized around 5 Å (Fig. 9a). The HSP90AB1-CDCA complex system reached equilibrium after 45 ns and stabilized around 3 Å. The EP300-CDCA complex system reached equilibrium after 10 ns and stabilized around 2.3 Å. The RMSD value of the EP300-CDCA complex system was the lowest, indicating that CDCA small molecules exhibit high stability when bound to EP300 target proteins.

Further analysis revealed that the Radius of Gyration (Rg) and solvent-accessible surface area (SASA) of the EP300-CDCA complex were more stable than those of the EP300-native ligand complex (Fig. 9b,c). This indicates that the target protein-small molecule complex remained stable and compact throughout the simulation. Hydrogen bonds are crucial for the binding of ligands to proteins. Kinetic analysis of hydrogen bonds revealed that the number of hydrogen bonds between the native ligand and the EP300 target protein ranged from 0 to 4, with the complex typically having around 2 hydrogen bonds. The number of hydrogen bonds between CDCA and the EP300 target protein ranged from 0 to 5 (Fig. 9d). In most cases, the complex exhibited approximately three hydrogen bonds, indicating strong hydrogen bond interactions between CDCA and the EP300 target protein.

Root mean square fluctuation (RMSF) quantifies the flexibility of amino acid residues in proteins. As shown in Fig. 2e, the RMSF value of the complex is relatively low (ranging from 1.7 to 2.2 Å), indicating low flexibility and high stability. In conclusion, the binding of CDCA small molecules to target proteins is stable, with strong hydrogen bonding interactions, indicating that CDCA binds effectively to target proteins.

### Effect of CDCA on lung pathology in model mice

**Fig. 10:**
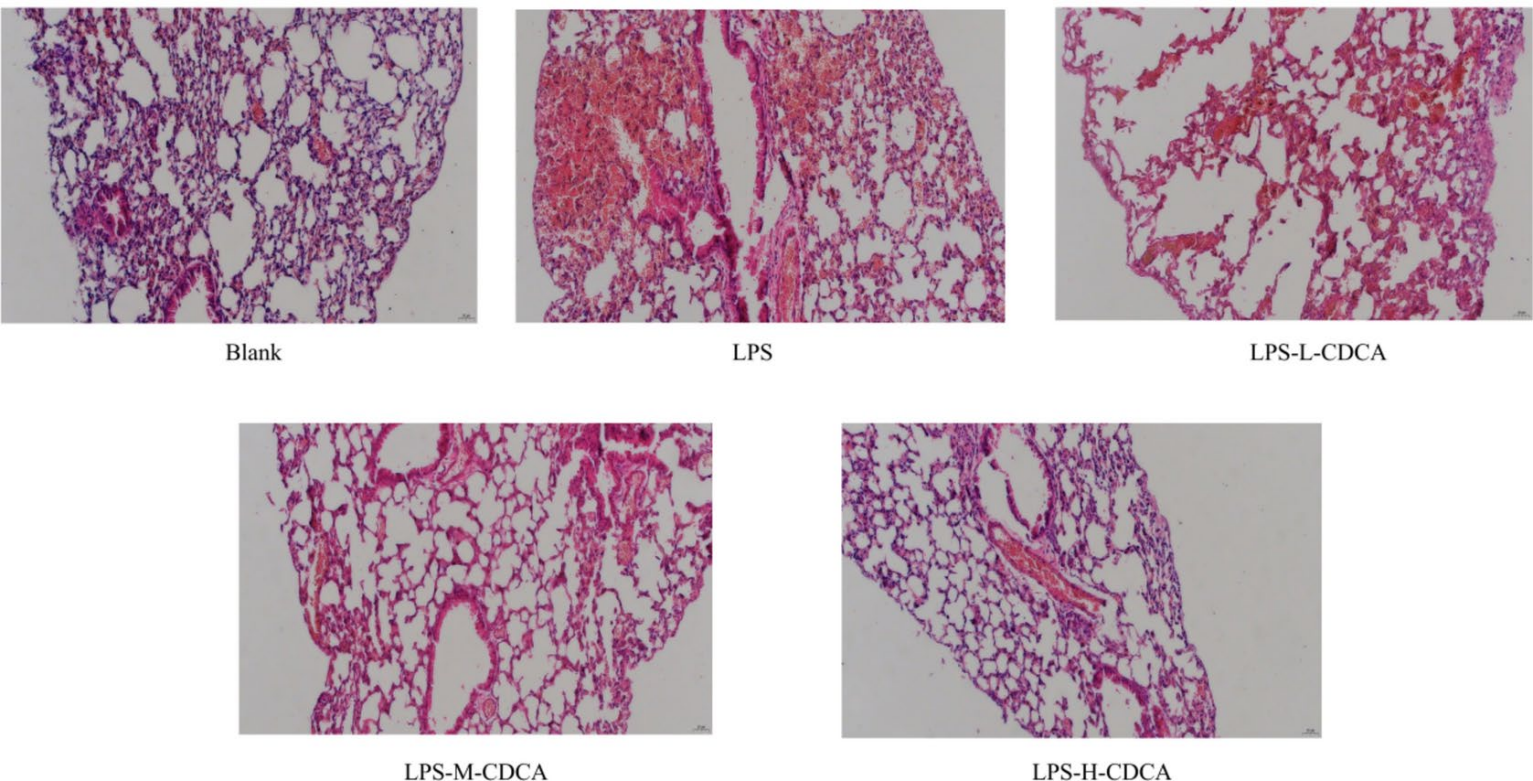
Lung tissue morphology of mice in each group.

Figure 10 illustrates the outcomes of HE staining in the lung tissues of mice across all experimental groups. Figure 10 reveals that the lung tissue morphology in the normal group exhibited normalcy, characterized by a clear and complete alveolar structure, with no significant inflammatory cell infiltration observed in either the alveoli or the lung interstitium. In the model group, lung tissue exhibited atrophy, disordered alveolar structure, significantly widened alveolar intervals, and a pronounced presence of inflammatory cells within both the alveoli and lung interstitium. Mild edema was observed, but congestion was absent in the lung tissues of mice in all CDCA dose groups; a minimal exudation of inflammatory cells was noted in the alveoli, along with widened alveolar intervals and lymphocyte infiltration surrounding the bronchioles and blood vessels. Notably, inflammatory cell infiltration was significantly reduced compared to the model group, with the most substantial reduction observed in the high-dose group.

### Effect of CDCA on serum inflammatory factors in model mice

**Fig. 11:**
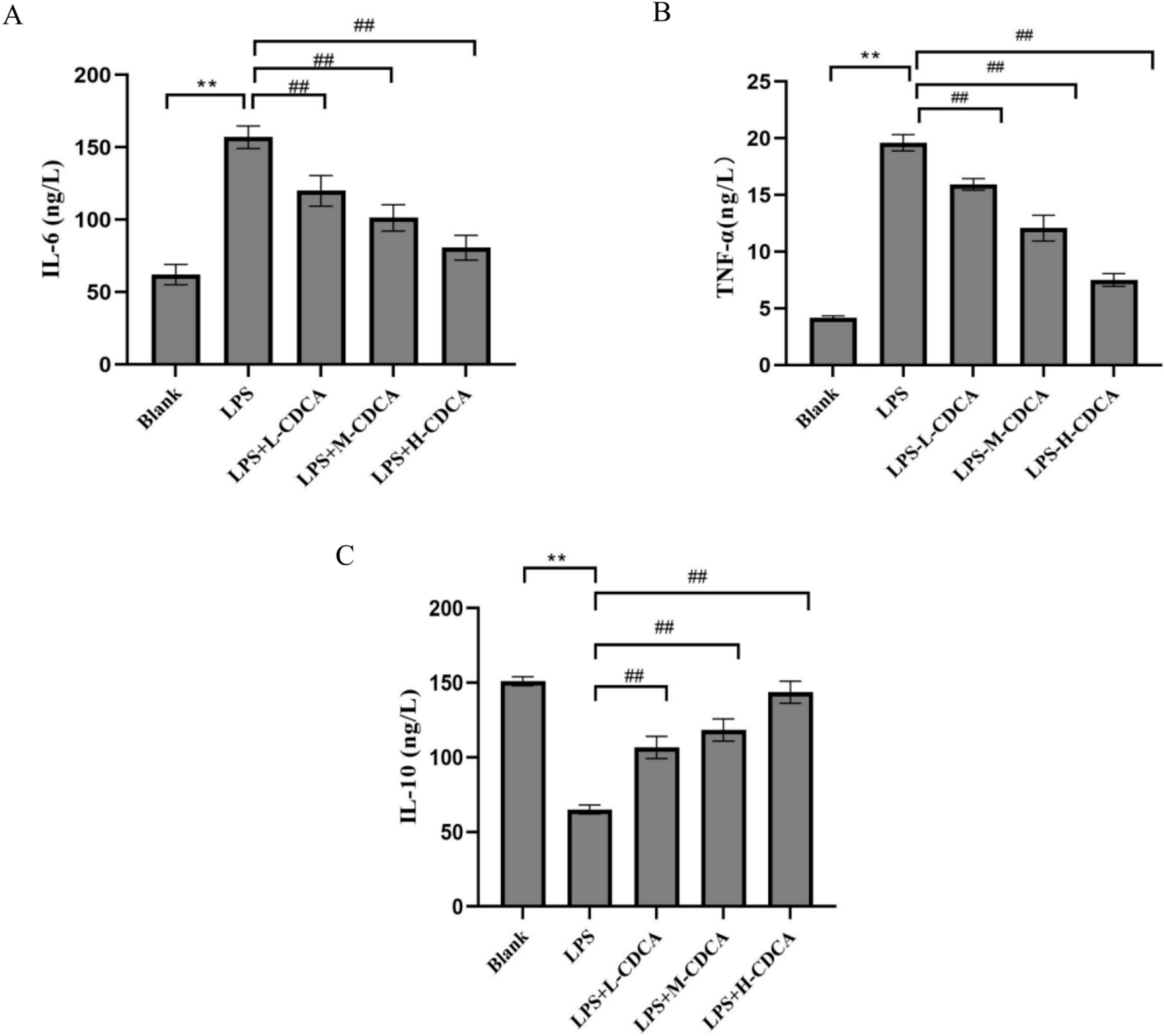
Serum IL-6, IL-10 and TNF-α contents of mice in each group. ** represents P < 0.01 compared with blank group, ## represents P < 0.01 compared with LPS group.

Figure 11 illustrates the serum cytokine content results across the various groups of mice. Figure 11 indicates that, compared to the normal group, the serum levels of interleukin-6 (IL-6) and tumor necrosis factor-alpha (TNF-α) in the model group were significantly elevated (P < 0.01), while the serum level of interleukin-10 (IL-10) was significantly reduced (P < 0.01). In comparison to the model group, the serum levels of IL-6 and TNF-α in the CDCA groups were significantly reduced (P < 0.01), while the serum level of IL-10 was significantly elevated (P < 0.01), particularly in the high-dose CDCA group (P < 0.01)**.**The results showed that CDCA could significantly reduce the serum levels of IL-6 and TNF-α and significantly increase the serum level of IL-10, thereby improving ALI in mice (P < 0.01).

### Effect of CDCA on the expression of related genes in lung tissue of model mice

**Fig. 12:**
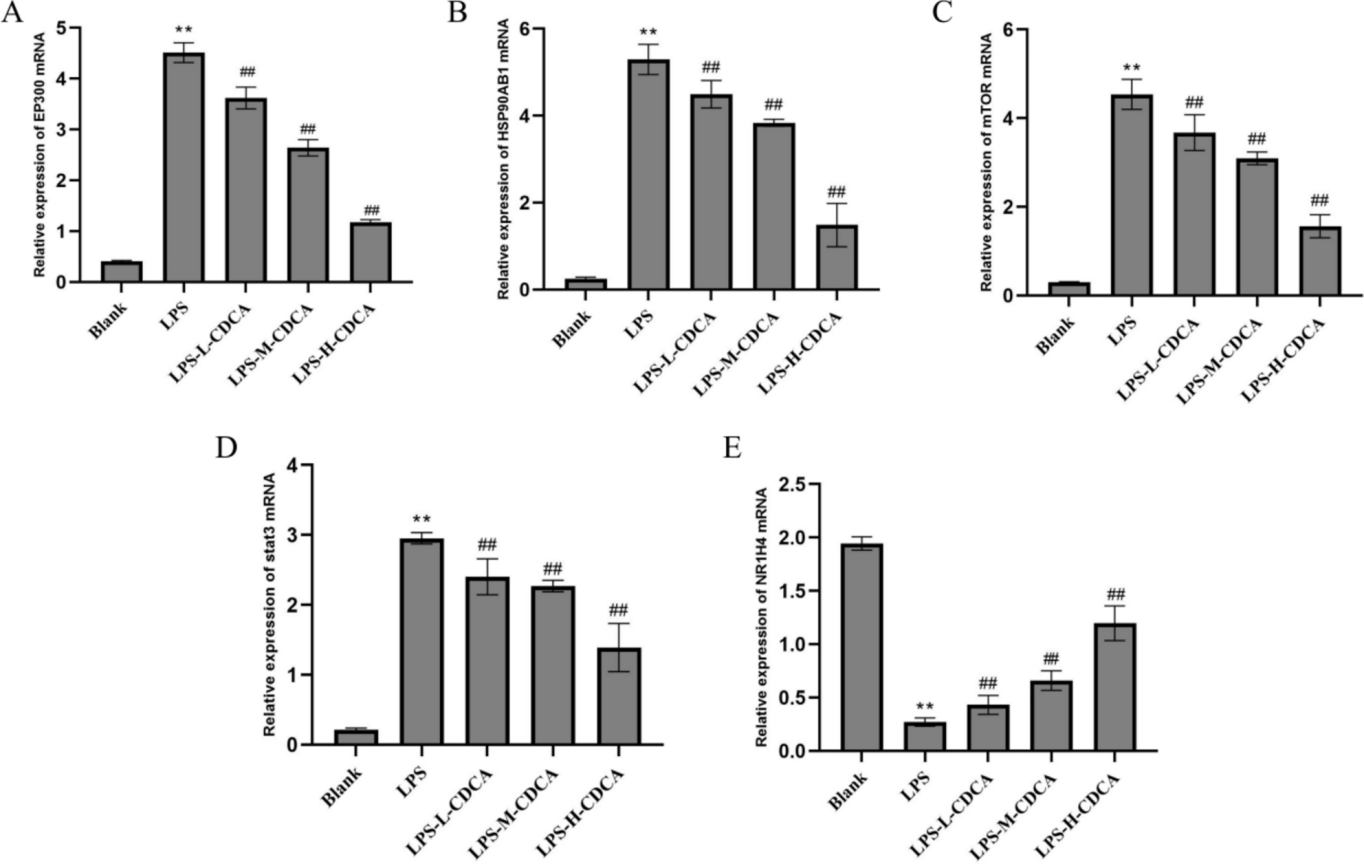
Regulatory effects of CDCA on key genes in mice. ** represents P < 0.01 compared with blank group, ## represents P < 0.01 compared with LPS group.

As depicted in Fig. 12, the expression levels of EP300, HSP90AB1, MTOR, and STAT3 mRNA in the lung tissue of the model group were significantly elevated compared to the normal control group, while the expression of NR1H4 mRNA was significantly reduced (P < 0.01). In comparison to the model group, the expression levels of EP300, HSP90AB1, MTOR, and STAT3 mRNA in the lung tissue of the CDCA groups were significantly decreased, while the expression of NR1H4 mRNA was significantly increased (P < 0.01). The results showed that CDCA could significantly reduce the mRNA expression levels of HSP90AB1, MTOR and STAT3 in lung tissue of ALI mice (P < 0.01), and significantly increase the mRNA expression level of NR1H4 (P < 0.01), thereby improving ALI.

## Discussion

Modern medical research has identified that ALI results from various direct and indirect injury factors, leading to diffuse pulmonary interstitial and alveolar edema, which subsequently causes acute hypoxic respiratory insufficiency. Pathophysiological features of ALI include reduced lung volume, decreased lung compliance, and a diminished blood flow-to-ventilation ratio. Patients typically present with progressive hypoxemia and respiratory distress. Lung imaging reveals heterogeneous exudative lesions. As the disease progresses to a severe stage, its clinical manifestations may become more pronounced^22–27^. Due to its rapid onset, severe condition, and high mortality rate, ALI poses a significant global public health challenge, with a current lack of effective treatments^28^. In this study, we systematically investigated the therapeutic potential of CDCA in the treatment of ALI through network pharmacology and molecular simulation techniques. Comprehensive network pharmacology analysis identified key targets of CDCA associated with ALI, spanning key biological processes integral to ALI pathophysiology, such as inflammation regulation, cell proliferation, apoptosis, and metabolism. The identified targets included HSP90AA1, STAT3, HSP90AB1, EP300, NFKB1, CD4, NR1H4, MTOR, and TLR4. To further validate the stability and specificity of these interactions, molecular simulation techniques were employed to simulate the binding processes of CDCA molecules to these target proteins, assess their binding affinity, and elucidate their binding sites. This analysis revealed how CDCA may inhibit or activate specific signaling pathways by forming stable complexes with these targets, thereby highlighting its potential therapeutic role in the treatment of ALI. Our findings largely align with prior studies while providing deeper insights into the mechanisms of CDCA action. Building on the findings of this study, CDCA has demonstrated therapeutic potential in treating ALI by targeting multiple key proteins. These targets are integral to ALI pathophysiology, and their significance has been confirmed in prior studies. Specifically, Qing et al. demonstrated that LPS-induced acute lung injury elevates HSP90 and NF-κB levels, and inhibition of these targets markedly ameliorates ALI symptoms^29^. The present study found that CDCA may mediate its therapeutic effects by targeting these proteins. Additionally, Qiang et al. showed that Nrf2 and STAT3 play pivotal roles in ferroptosis by regulating SLC7A11, mitigating ALI-associated pathological processes^30^. CDCA may similarly modulate ALI pathology through STAT3 and related targets. Liu et al. reported that colchicine suppresses STAT3 phosphorylation via STAT3/EP300 interactions, inhibiting NLRP3 promoter acetylation and ameliorating sepsis-induced ALI^31^. This study confirms EP300 as a crucial target of CDCA and suggests that CDCA may act through a similar mechanism. Studies have also shown that CD4 (+) T cells are essential for lung defense but can exacerbate inflammation during lung infections^32^. CDCA may regulate the inflammatory response in ALI by modulating CD4 (+) T cell function. Similarly, Wu et al. demonstrated that airway diseases inhibit MTOR, enhancing autophagy and impairing lysosomal activity^33^. The present study identified MTOR as a key CDCA target, suggesting that CDCA may exert therapeutic effects by modulating MTOR and associated autophagy mechanisms. Finally, Chen et al. showed that silencing TLR4 reduces LPS-induced liver injury by inhibiting the TLR4/MyD88/NF-κB signaling pathway^34^. This study also identified TLR4 as a core target of CDCA, indicating that CDCA may inhibit TLR4 and downstream pathways to exert anti-inflammatory effects. In conclusion, CDCA exerts biological effects in ALI by targeting proteins such as HSP90, NF-κB, STAT3, EP300, CD4, MTOR, and TLR4. To further verify whether CDCA acts through these targets, we examined the expression levels of related genes in animal models and found that CDCA improved ALI by regulating these genes, which aligns with the results of molecular simulations. These findings suggest that molecular simulations are a powerful tool for predicting the relevant targets through which CDCA improves ALI. These targets are integral to key processes like inflammation regulation, cell proliferation, apoptosis, and metabolism. By modulating these targets, CDCA demonstrates therapeutic potential for ALI treatment. This finding provides a theoretical basis for CDCA’s clinical application and offers novel insights for future drug development.

Network pharmacology and molecular docking are research methodologies grounded in systems biology and network theory, offering innovative approaches to drug research and development. By constructing drug-target-disease networks and analyzing the interactions between drugs and diseases, these methods provide new perspectives in understanding drug mechanisms. In clinical research, network pharmacology and molecular docking are extensively applied in areas such as pharmacodynamic substance discovery, mechanism of action exploration, safety evaluation, personalized medicine, and the identification of new indications for traditional Chinese medicine prescriptions. Through network pharmacology, the active ingredients in Chinese medicine prescriptions can be identified, and their interactions with disease-related targets can be analyzed to reveal the pharmacodynamic material basis of these prescriptions. Constructing drug-target-disease networks, analyzing the interactions between drugs and diseases, and identifying key nodes and regulatory pathways facilitate the understanding of therapeutic pathways and the regulatory networks involved. XIONG et al.^35^ conducted a study on the screening of potential active ingredients from Smilax glabra Roxb. and their key targets and associated pathways for the treatment of metabolically related fatty liver disease using network pharmacology and molecular docking techniques. In vivo experiments in zebrafish demonstrated that SGR intervention significantly reduced lipid accumulation, alleviated oxidative stress, and regulated the composition of the gut microbiota. These findings suggest that network pharmacology and molecular simulation techniques are effective tools for screening potential drug candidates.

Previous studies have confirmed that the massive release of inflammatory mediators, triggered by pathogen-related antigens on the cell membrane of lung tissue in response to LPS, a component of the cell wall of gram-negative bacteria, constitutes the principal mechanism underlying the occurrence and progression of acute lung injury^36^. Effectively inhibiting the release of inflammatory mediators and delaying the progression of ALI has consistently been a focal point of medical research. In cases of severe COVID-19 infection, a cytokine storm may significantly impair lung function and exacerbate disease severity. Clinical studies have identified elevated concentrations of pro-inflammatory factors, particularly TNF-α and IL-6, in the serum of COVID-19 patients^37^. Excessive recruitment of neutrophils in lung tissue, however, may exacerbate cytokine storm formation and blood embolism, resulting in lung inflammation and injury^38^. We employed an LPS-induced ALI mouse model to investigate the anti-ALI effects of CDCA. Research indicates that CDCA can ameliorate pulmonary edema associated with LPS-induced acute lung injury in mice, diminish telangiectasia and congestion within lung tissue, attenuate inflammatory cell infiltration in the trachea, and decrease the proliferation of fibrous tissue in the lung interstitium, along with significantly reducing serum levels of the inflammatory factors IL-6 and TNF-α. Consequently, it may contribute to the treatment of ALI.

This study employed network pharmacology and molecular simulation techniques to elucidate the potential mechanism of CDCA in treating ALI, supported by validation through animal experiments. While this study provides valuable insights into the therapeutic potential of CDCA for ALI, several limitations must be acknowledged. First, the reliance on network pharmacology and molecular simulation techniques introduces a level of uncertainty due to the inherent limitations of the databases and algorithms used. Incomplete or inaccurate data from public databases may impact the reliability of predicted targets and pathways. Additionally, molecular simulation methods, while powerful, depend heavily on algorithmic assumptions that may not fully capture the complexity of biological interactions. Second, the validation of CDCA’s efficacy was limited to animal experiments, which, although useful, do not fully replicate human physiology. Species-specific differences in metabolism, immune responses, and molecular pathways may result in discrepancies between animal models and clinical outcomes. Furthermore, this study did not explore potential off-target effects or the pharmacokinetic profile of CDCA, both of which are critical for understanding its safety and efficacy in humans. Lastly, the study did not address the potential for combinatory effects with other therapeutic agents or the long-term consequences of CDCA treatment, leaving gaps in its applicability to real-world clinical scenarios. Future research should prioritize conducting large-scale clinical trials to assess the safety, efficacy, and optimal dosage of CDCA, alongside its pharmacokinetics and potential side effects. Mechanistic studies using advanced techniques, such as single-cell RNA sequencing and proteomics, are essential to elucidate how CDCA modulates key signaling pathways in ALI. Long-term safety evaluations are needed to assess immune impacts, toxicity, and chronic risks, complemented by the refinement of biomarkers for precise therapeutic monitoring. Additionally, systematic exploration of off-target effects using in silico and experimental methods will ensure CDCA’s safety and efficacy as a therapeutic agent for ALI.

## Conclusion

In this study, we systematically explored the mechanistic basis of CDCA in the treatment of ALI using network pharmacology and molecular simulation techniques. We identified several key targets, including HSP90AA1, STAT3, HSP90AB1, EP300, NFKB1, CD4, NR1H4, MTOR, and TLR4, which are integral to ALI pathophysiology. Using molecular simulation techniques, we further validated CDCA’s stable binding to these targets and examined their potential interaction modes. Animal experiments demonstrated that CDCA significantly ameliorated lung tissue pathology in LPS-induced ALI mice, regulated cytokine levels, and modulated the mRNA expression of related targets. Although the reliability of computational analysis is constrained by database integrity and algorithmic accuracy, and the findings from animal experiments require further validation through clinical trials, this study offers a theoretical foundation and novel insights for CDCA’s clinical application in ALI treatment. Future research should focus on elucidating the precise mechanisms underlying CDCA’s therapeutic effects in ALI and conduct large-scale clinical trials to facilitate its clinical implementation.

## Data Availability

The datasets used and/or analysed during the current study available from the corresponding author on reasonable request.
